# Dr. George Rite Kinne

**Published:** 1914-06

**Authors:** 


Dr. George Rite Kinne, Syracuse, 1876; died at his home in Syracuse, April 15, aged 60.

